# Emergency remote teaching evaluation of the higher education in Indonesia

**DOI:** 10.1016/j.heliyon.2021.e07788

**Published:** 2021-08-16

**Authors:** Ani Cahyadi, Hendryadi, Sri Widyastuti, Vika Nurul Mufidah, Achmadi

**Affiliations:** a Tarbiyah and Teacher Training Faculty, Universitas Islam Negeri Antasari, Banjarmasin, Indonesia; b Sekolah Tinggi Ilmu Ekonomi Indonesia Jakarta, Jakarta, Indonesia; c Economics and Business Faculty, Universitas Pancasila, Jakarta, Indonesia; d Islamic Religion Faculty, Universitas Nahdlatul Ulama Indonesia Jakarta, Jakarta, Indonesia; e Sekolah Tinggi Ilmu Ekonomi Tunas Nusantara, Jakarta, Indonesia

**Keywords:** Emergency remote teaching, Student-centered, COVID-19, Humanizing pedagogy, Indonesia, Developing countries

## Abstract

The present study introduces the qualitative and quantitative results obtained in a pilot study of emergency remote teaching (ERT) evaluation in Indonesian higher education. In particular, this study aims to provide basic principles for future ERT implementation. Seven universities in Indonesia were involved in the first phase of the study, aiming to obtain initial information regarding the relevance, content validity, and readability of fundamental initial principles of ERT. The second phase aims to assess the quality ERT scale quality using confirmatory composite analysis (CCA) procedure, involving 2,957 undergraduate students from 22 universities in ten provinces in Indonesia. The results seem to indicate that the ERT principles can be viewed as 1) a complementary tool for ERT design covering five principles including simplicity, accessibility, affordability, flexibility, and empathy; and 2) In Indonesian higher education, affordability and flexibility are the two principles that are rated the lowest based on students' experiences. The lack of access to a fast, affordable, and reliable Internet connection in some areas of Indonesia is a fundamental problem in implementing ERT. This result is likely to be the case in other developing countries with similar geographical characteristics. The findings provide information based on practical experience - showing that learning design in a crisis is dynamic and open to revision based on socio-economic considerations, technological infrastructure, and students 'and teachers' readiness.

## 1 Introduction

The education sector is one of many areas that have been affected by the COVID-19 pandemic. After Indonesian President Joko Widodo announced that it was a national epidemic, all organizations were instructed to implement large-scale social distancing to prevent the spread of COVID-19, including in the education sector. In line with the presidential instruction, the Indonesian minister of education and culture announced national exams for all levels(elementary, junior, and high school) on 24 March 2020 (Ghaliya, 2020). Furthermore, since March 2020, all education institutions were instructed to prepare for implementing emergency remote teaching (ERT) modes as a quick solution for sustainable education by minimizing transmission risk.

Emergency remote teaching is defined as a sudden interim shift of instructional delivery from face-to-face to an online delivery mode as a result of a disaster/crisis. ERT is contrary to online learning, which is pre-planned and designed to be delivered virtually (Hodges et al., 2020). The main purpose of ERT is not to completely transfer the conventional methods to e-learning, but to provide temporary access during emergencies using various available and reliable media or platforms. Thus, ERT can be understood as a temporary solution and should be separated from the term “online learning” (Hodges et al., 2020). Accordingly, online learning is an alternative and flexible option for universities/colleges, while emergency remote teaching is an obligation to protect the educational community from spreading the virus. Therefore, online learning and emergency distance teaching are not the same since their purpose and function are different.

ERT is an obligation and a realistic solution during crisis circumstances. Several authors had a different research focus in highlighting ERT activities, such as differences between online learning and ERT, emergency curriculum design, and how to evaluate ERT. For example, Hodges et al. (2020) specifically provide important points regarding the difference between ERT and online learning. Mohmmed et al. (2020) evaluated ERT implementation. Wang and East (2020) constructed an emergency curriculum during the pandemic, while Whittle et al. (2020) developed a conceptual framework for responsive online teaching in a crisis. Other studies (e.g., Green et al., 2020; Karakaya, 2020) used the activity-centered analysis and design (ACAD) framework to design ERT in New Zealand, and Karakaya (2020) focused on a human-centered approach. Several researchers have focused on various pedagogical constraints in remote teaching activities (Bozkurt and Sharma, 2020; Ferri et al., 2020; Gelles et al., 2020; Peters et al., 2020; Rapanta et al., 2020). More comprehensively, Reimers et al. (2020) developed a module as a framework to guide an education response to the COVID-19 Pandemic. This module aims to support education leaders in developing three essential components: curriculum, professional resources, and tools or technology used as learning media. However, none of them specifically developed principles for implementing ERT. In addition, geographical differences and different technological readiness between countries cause the conditions for ERT implementation to be different, thus giving the possibility of different results. Thus, this study aims to fill this gap by proposing the principles for implementing ERT more universally.

This study combined the principles of online learning, including flexibility, accessibility, affordability, and development of a robust educational ecosystem (Hodges et al., 2020), and the three principles of simplicity, flexibility, and empathy (The University of Auckland, 2020). The current study examined administrators', lecturers', and students' perceptions as reflections of the principles and challenges behind the implemented ERT model based on the current situation. ERT principles are novel in their foundational work and guide developing online courses and distance learning modes in unplanned or responsive remote teaching situations. Specifically, this study explored two research questions: (1) What are the main principles for implementing ERT?; (2) How do students' perceptions about emergency remote teaching during the pandemic? Two studies with a mixed-method approach were conducted to answer these questions: the first study to explore the relevant principles in implementing ERT involving an expert panel for content validity, readability, and logical flow. The next phase (Study 2) is a quantitative endeavor involving 2,957 undergraduate students from 22 universities in ten Indonesian provinces. This second stage provides an overview of student responses to the five principles generated in Study 1.

## 2 Literature review

### 2.1 ERT and human-centered pedagogy

In ERT, learning must be designed quickly by utilizing existing resources to continue the educational process. However, it is undeniable that the response of educational institutions is almost the same and that they are experiencing a shock in response to the COVID-19 pandemic, mainly because of societal shifts (Aguliera and Nightengale-Lee, 2020). While all countries ordered all provinces, districts, and school levels to close face-to-face learning services in schools and shift activities to online media, the response was mixed. The speed of the change from conventional methods to online delivery was staggering (Hodges et al., 2020), especially for institutions that were not adequately prepared in terms of technology and human resources. In short, learning must be designed through the innovations of each university and teaching staff to develop an effective ERT. Because ERT is a temporary solution, from a practical point of view, it should be distinct from the concept of online learning (Hodges et al., 2020), which requires a series of development processes.

The essence of ERT design is the ability of universities and educational institutions to understand internal conditions (teachers' ability to manage Internet-based learning) and external conditions (students and society) in order to find the best way that can be accepted by all parties (Robinson et al., 2017). For example, in the COVID-19 pandemic situation, both teachers and students may experience mental and physical health issues, so the ERT must be designed to be as flexible as possible to accommodate learners’ and teachers' lived experience to reduce unnecessary stress (Price and Osborne, 2000; Shelton et al., 2020).

Human-centered pedagogy is an approach that must be adopted to design student-centered learning (Karakaya, 2020; Luka, 2014). The term “humanizing”, referring to Freire's philosophy, focuses on "the cognitive capacity of humans to shape their experiences and achieve personal and collective self-actualization, thus developing their full humanity" (Salazar, 2013). From an educational perspective, this idea was developed more specifically as a cognitive approach to becoming more reflexive in dealing with issues of power, access, and representation (Luka, 2014). Previously, Price and Osborne (2000) described humanizing pedagogy as a pedagogy that focuses on developing the entire person. Thus the reliance on humanizing pedagogy as the basis for ERT is aimed more at providing valuable experiences for learners and their well-being (Salazar, 2013). In other words, in an emergency, universities and teachers cannot obsessively focus on teaching delivery, knowledge transmission, and lectures using various tools or sophisticated technology (Bozkurt and Sharma, 2020), but must instead focus on care, collaboration, support, and empathy (Wang and East, 2020).

### 2.2 Prior studies of emergency remote teaching principles

Emergency remote teaching, as the name implies, is a learning method specifically designed for emergencies. Using this understanding, ERT is not a long-term solution but a temporary one to maintain educational sustainability. Several authors have focused on remote teaching mode in special situations. For example, Mohmmed et al. (2020) evaluated ERT implementation. Through a case study at Middle East College in Oman, the authors used the CIPP (Context, Input, Process, Product) evaluation model to assess the effectiveness of the adopted learning model during COVID-19. This study provides recommendations for implementing asynchronous activities to provide additional opportunities and flexibility for students compared to synchronous mode. Wang and East (2020) highlighted Chinese curriculum decisions during emergency remote teaching in the context of higher education in New Zealand. They noted that ERT was designed to be student-centered, communicative, and task-based. This approach is considered to build experiential learning and build a strong rapport with students through hands-on activities and teamwork in class. Also, pedagogical innovations are key to the successful implementation of an emergency curriculum.

Whittle et al. (2020) developed a conceptual framework for responsive online teaching in a crisis. They introduced an emergency remote teaching environment (ERTE) framework, which consists of the following stages: inquiry, classification, design, and finally evaluation. Their study highlights several important aspects of design, learning goals that must be adapted to the situation, paying attention to the ratio between lecturers and students, developing good communication, and encouraging students to become agents of learning in online education. Teachers discussed the possibility of learner-driven assignments. Other studies (e.g., Green et al., 2020; Karakaya, 2020) used the activity-centered analysis and design (ACAD) framework to design ERT in New Zealand, and focused on a human-centered approach. Green et al. (2020) used a theoretical approach to design a transition during an emergency in New Zealand. They drew on the ACAD framework and discussed educational design implications, providing a detailed explanation of tools, social arrangements, and tasks to support learning activities in emergency remote education. On the other hand, Karakaya (2020) gave special notes on the approach of humanizing pedagogy and the pedagogy of care as part of student-/learning-centered design (see Baran and AlZoubi, 2020; Bennett et al., 2017; Luka, 2014; Salazar, 2013).

There is a consensus among researchers that ERT should be treated as a temporary solution to an immediate problem and calls for the promotion of simplicity, flexibility, and empathy (Mohmmed et al., 2020; Rapanta et al., 2020; The University of Auckland, 2020). On the other hand, online learning is optional and is best described as an innovation to increase flexibility, accessibility, affordability, and the development of a robust educational ecosystem (Hodges et al., 2020). We argue that it is not sufficient to base ERT solely on the three principles of simplicity, flexibility, and empathy (Mohmmed et al., 2020). It is also necessary to consider two additional aspects, accessibility, and affordability, based on community Socio-economic considerations and differences in Internet access speed, which often become obstacles, particularly in developing countries. Therefore, we propose five principles—simplicity, accessibility, affordability, flexibility, and empathy—by combining the previous works (Hodges et al., 2020; Mohmmed et al., 2020; The University of Auckland, 2020).

Simplicity refers to a simple, uncomplicated, and free from complexity. ERT should fulfill this principle based on the nature of ERT's as a temporary solution so that it does not mean to transfer conventional methods to e-learning completely. Accessibility refers to making learning delivery usable by as many people as possible. Thus, teachers can choose applications and online learning media that are easy to access to overcome the disparities in the capacities and capabilities of electronic devices used by students (Ferri et al., 2020). Empathy refers to the attention, motivation, care, and a sense of belonging from teachers to students in online courses amid a crisis. The teachers to carry out pedagogical innovations to increase student engagement by creating a climate of empathy and care (Bozkurt and Sharma, 2020) and providing motivation, and showing a sense of belonging for students in online courses (Wang and East, 2020). Flexibility is the priority in order to address the learning process and outcomes. Although moving to online instruction can enable the flexibility to teach anywhere and anytime without having face-to-face meetings, the speed required to change from conventional methods to online is real and staggering (Hodges et al., 2020). Affordability refers to being cheap enough for people to be able to buy or student can afford to pay fees related to online learning (Hodges et al., 2020).

## 3 Materials and methods

This study uses a mix-methods approach to obtain comprehensive information about the ERT principles through opinions from teachers, administrators, and students at universities in Indonesia to capture the current situation and various inputs related to the five proposed principles. The first stage of the study aims to explore the initial principles. We involved seven universities in Indonesia in obtaining initial information regarding relevant factors that can be used as a basis for evaluation in implementing ERT during the pandemic. Study 1 was conducted for three months (July to September 2020). The second stage study aims to measure the five ERT principles generated in Study 1 by distributing online questionnaires to 2957 undergraduate students in ten provinces in Indonesia. Data collection was carried out starting from the 25th March to 2nd April 2021.

We argue that this approach is well suited for this study for two reasons. First, this methodological approach balances the qualitative and quantitative approach of proposed ERT principles with the emergent needs of participants. Second, data collection in crisis contexts can be highly unstructured and unpredictable (Lin et al., 2017). This situation allowed us to acknowledge the novelty of the ERT phenomenon quickly rather than gathering extensive data that would entail a long time to validate the theories. We conceive the principles from the first study and follow-up questionnaires to obtain opinions on the principles as intertwined. The next phase (Study 2) is a quantitative endeavor involving 2,957 undergraduate students from 22 universities in ten Indonesian provinces. This second stage provides an overview of student responses to the five principles generated in Study 1. Ethical approval was obtained from the Tarbiyah and Teacher Training Faculty, Universitas Islam Negeri Antasari. Respondents’ participation was completely consensual, anonymous, and voluntary. The collecting data was conducted according to the Declaration of Helsinki.

Study 1 The Study 1 was conducted in two phases. First, we engaged a panel of seven experts in the initial design of ERT principles. To broaden the scope as much as possible, we included two practitioners from the field of educational technology and five senior lecturers from different universities. The five principles proposed based on the literature review included simplicity, flexibility, empathy (The University of Auckland, 2020), accessibility, and affordability (Hodges et al., 2020). For content validity, the expert panel was asked to provide input on the items' appropriateness and relevance to the Indonesian education context. Furthermore, the expert panel provides an assessment of the relevance of items with 4 Likert types of items ranging from "relevant = 4" and "irrelevant = 1." This stage produces five relevant principles as the basis for evaluating ERT implementation, including simplicity, accessibility, affordability, flexibility, and empathy (See Table 1).

**Table 1:** The summary of emergency remote teaching principles.

The ERT Principles	Source
• Simplicity • Flexibility • Empathy	The University of Auckland (2020); Wang and East (2020); Mohmmed et al. (2020)
• Flexibility • Accessibility • Affordability	Hodges et al. (2020)
• Empathy	Bozkurt and Sharma (2020)

After the five ERT principles were identified, we invited 21 respondents, including the College Dean, Associate Dean of Academics, and faculty quality assurance from seven universities in Indonesia. All participants were asked for their opinions regarding simplicity, accessibility, affordability, flexibility, and empathy in the previous phase. All qualitative data were reviewed and analyzed to identify emerging themes and patterns. Qualitative data through an open-ended questionnaire were analyzed using the triangulate approach (Creswell and Clark, 2017).

Study 2 The second study was conducted using a quantitative approach by distributing questionnaires to 22 universities in ten provinces in Indonesia. Data were collected through an online questionnaire through representatives of the universities involved in this study. The sampling method uses purposive and snowball techniques. The faculty members from universities/colleges involved in this survey have responsible for collecting data on behalf of their campus. After obtaining approval from the institution, the faculty representatives distributed the questionnaire link through the Whatsapp group at the faculty and student communities. Participation is voluntary and anonymous to obtain objective responses from students.

The online questionnaire was distributed via the Whatsapp group between 25^th^March to 2^nd^April 2021. A total of 3,019 responses were received, but 62 responses were eliminated because they did not complete all the questions and biographical information. Finally, 2,957 responses were used for further analysis. A summary of the collected data with regards to type university (private - state university), gender, age, and employment is shown in Table 2 as a summary of general information of respondents, The questions measuring the variables were based on well-established measurements derived from Study 1. All items were rated on a 5-point Likert scale ranging from 1 (strongly disagree) to 5 (strongly agree).

**Table 2:** Content validity and item description.

	Statement of items	Mean	CVI	S.D
Simplicity	Ease of use of applications used in learning: The learning media used by the teacher is user friendly	3.57	0.89	0.53
Accessibility	Ease of access: the applications used in online learning is easy to access in any location and anytime	3.71	0.93	0.49
Affordability	Applications used in cheap learning (in terms of internet quota usage);	3.57	0.89	0.53
Flexibility	Teachers provide high flexibility concerning material delivery through video recordings that can be accessed anytime and anywhere by students.	3.71	0.93	0.49
Empathy	The Teacher provides attention, motivation, care, and a sense of belonging for students in online courses	3.86	0.96	0.38

Notes: CVI = content validity index; S.D = standard deviation; n = 7.

We used confirmatory composite analysis (CCA) to test the quality of the ERT principles scale. The CCA was chosen because it has practical implications for both principal components analysis and common factor analysis (Hair et al., 2020). Also, this technique offers an alternative approach to validating the measurement models, helpful in developing new measures, and can be used in both exploratory and confirmatory measurement models. We followed the six CCA analysis procedure based on the recommendations of Hair et al. (2020): "1. estimate the loadings and significance; 2. assess indicator reliability (items); 3. assess composite reliability (construct); 4. determine the average variance extracted (AVE); 5. assess discriminant validity; 6. determine nomological validity " (p. 104). For the purposes of the phase 6 test, we used a comparison scale of 5 items technology integration and application (TIA) adapted from Chang et al. (2015) with permission.

## 4 Results

### 4.1 Study 1 results

The first stage of the study answers the question: What are the main principles for implementing ERT? We use the content validity index (CVI) to assess the feasibility of an item. The item will be considered relevant if above the cut-off value of 0.80 (Polit and Beck, 2006). Table 1 displays the CVI numbers on five principles ranging from 0.86 - 0.96 and the mean rating score of 3.43–3.86, which meets the content validity standard (Polit and Beck, 2006).

#### 4.1.1 Simplicity

The main objective of ERT is to keep learning functioning and feasible for remote teaching without overburdening students, teachers, and parents during a crisis (Bozkurt and Sharma, 2020; Wang and East, 2020). In order for it to be a simple system, the design and implementation of ERT are related to curriculum specifically designed for emergency situations. Adopting an emergency curriculum enables teachers to respond quickly to maintain educational sustainability and ensure that students are supported in achieving their learning goals in a difficult situation. ERT can use various available and reliable media or platforms. ERT is neither an attempt to fully teach the study material in an online mode using various “advanced” applications nor a time to strive for the “best practice” in online delivery (Wang and East, 2020). Instead, ERT is focused on delivering practical learning with quick and simple approaches to online delivery of materials and assignments. ERT is not intended to meet learning objectives and standards in normal times, but rather to provide convenience by reducing basic competencies and study subjects.

As the frontline in educational systems, teachers need an emergency curriculum to help them make decisions about implementing learning activities and evaluating student outcomes (Wang and East, 2020). The following is a comment from a lecturer:

“Not reducing the material, just trying to tighten the learning hours, the duration is made not burdensome for students by adding more flexible discussion sessions after the delivery of the material.”

#### 4.1.2 Accessibility

Accessibility refers to making learning delivery usable by as many people as possible. Thus, teachers can choose applications and online learning media that are easy to access to overcome the disparities in the capacities and capabilities of electronic devices used by students (Ferri et al., 2020). Other difficulties that arise with ERT include the lack of an online teaching infrastructure. Problems also arise regarding the information gap, the complex environment at home (for example, having to share a device with family members or families being exposed to the virus), and so forth (Zhang et al., 2020). The following is a comment from a lecturer:

"We realize that not all students have a laptop to take online classes. Most of them only rely on smartphones, which have many limitations when it comes to assignment activities. On the other hand, students cannot freely interact directly with their peers like normal times. We agree that learning activities now focus more on easy and broad access by all students. "

We found that most respondents reported Internet speed instability as a significant issue for lecturers and students. Applications such as WhatsApp, Google Classroom, Google Meet, and Zoom were used in combination as learning delivery media by the majority of respondents. These applications were chosen based on considerations of accessibility, convenience, and, in general, ease of use by both lecturers and students. Thus, the learning delivery method met the principle of accessibility, and lecturers creatively innovated in delivering material by combining video recordings, modules, and PowerPoints.

#### 4.1.3 Empathy

In an emergency, teachers are expected to carry out pedagogical innovations to increase student engagement by creating a climate of empathy and care (Bozkurt and Sharma, 2020), as well as providing motivation and showing a sense of belonging for students in online courses (Wang and East, 2020). Students should know where to find support from teachers and the campus in remote teaching during a crisis. One lecturer expressed how they felt about the empathy shown during emergency teaching:

“We heard that many of our students have been exposed to COVID-19. There are some of our students who take online study and exams while being treated at the hospital. However, I only got this information when the student had recovered. I am grateful and proud of the students' enthusiasm for learning.”

Another effort by faculty is to provide information and a complaint center. Some universities provide a 15% discount on tuition fees, waivers for tuition fees, and even full scholarships. They also provide assistance with Internet quota fees and other policies to ensure that students in poor financial situations can continue their studies. ERT focuses more on the teacher's efforts to execute the learning function and is feasible for remote online delivery without increasing stress among students and teachers during difficult times (Wang and East, 2020). A lecturer gave the following opinion:

“We are fully aware that many families have been directly affected by the pandemic, such as the inoperability of the business sector, reduced salaries, and even job cuts experienced by parents of students. I always give messages to lecturers to actively ask about the conditions of students, and continue to maintain student learning motivation. So far, I have heard that lecturers have a direct connection with student groups through WhatsApp groups, so that any information can be easily discovered by the lecturer.”

Finally, we asked them two questions about the effectiveness of ERT in terms of student interactions and achievement of learning material, and to choose whether they preferred online or face-to-face. Although more than 50% of respondents stated that achieving the material and interacting with students went well, more than 65% would prefer face-to-face learning post-pandemic. A limitation of technology resources is that obstacles are encountered during online tutorials with low-speed and unstable Internet networks in several regions of Indonesia. This emphasizes that the application of distance learning in Indonesia still requires time and in-depth evaluation before being widely implemented.

#### 4.1.4 Flexibility

Some universities provide high flexibility concerning material delivery through video recordings that can be accessed anytime and anywhere by students. However, in terms of lecture time, students are still required to attend virtual classes according to predetermined hours and days. We fully agree with this step, understanding that the pandemic situation decreased relatively in November 2020 (in some parts of Indonesia, some schools were even given permission to conduct face-to-face classes while following the health protocol from the government). A senior lecturer stated:

“We try to be flexible as possible to make it easy for students. We understand that the current situation is unfavorable, and many of us have also been directly affected by this pandemic. But we also keep our virtual meetings on schedule to monitor the student's condition, I think this is the best way we can do it.”

The pandemic crisis has changed the context of society, education, the economy, and individuals. From the perspective of complexity theory, systems are unpredictable and organizations must be able to continue to interact and obtain accompanying feedback on what to do while considering the social and organizational changes. Thus, the implementation of ERT needs to emphasize shared responsibility among faculty members and support staff, and requires a collective decision by all participant groups (including students) rather than “a centrally managed plan” (Wang and East, 2020). .

#### 4.1.5 Affordability

Affordability refers to being cheap enough for people to be able to buy or student can afford to pay fees related to online learningIn this context, students whose families have economic problems due to COVID-19 are a major concern. Using a virtual class/videoconference with a synchronous mode, apart from requiring fast Internet access, can also consume Internet data. Most Indonesian Internet users rely on expensive limited-capacity mobile networks (Harto, 2020), which makes it difficult for students to use broadband networks to meet their online learning requirements. For example, video conferencing uses large volumes of data.

We appreciate the work of lecturers who have a sense of social responsibility to not impose certain applications as mediums for delivering the material. The majority of respondents stated that the use of certain applications needs to be discussed with students to ensure that the learning process can run efficiently. Thus, learning delivery can use a mixture of synchronous and asynchronous environments based on the evaluation of the situation.

### 4.2 Study 2 results

Study 2 is to evaluation the ERT principle scale's quality using the CCA technique. The results of 6 stages of CCA are as follows:

**Step 1–2**: assessing the indicator loadings and indicator reliability. The standardized loadings in the analysis were in the range 0.75–0.84 (See Table 2). All items with standardized loadings above 0.70, as recommended by Hair et al. (2020). This outer loading evaluation indirectly specifies the indicator reliability and completes the second step (*p*-value < 0.05).

**Step 3**: the construct reliability is evaluated via the Cronbach's alpha (CA) and composite reliability (CR) values. This analysis shows that the α value 0.87 and CR 0.91, which is higher than the cut-off value of 0.70 (Hair et al., 2010, 2020).

**Step 4:** Convergent validity is evaluated using the average variance extracted (AVE) value. The AVE is 0.66, which is greater than the cut-off value of 0.50 (Hair et al., 2020).

**Step 5–6**: Discriminant validity is evaluated with the Fornell – Larcker criterion (Hair et al., 2020). The correlation of the ERT constructs with other variables (TIA). As shown in Table 2, AVE's square root value is 0.81 over the correlation between the variables (r = 0.76). Thus, convergent and discriminant validity has been confirmed. The correlation between ERT principles and technology integration and application (TIA) is also used as a stage 6 test (nomological validity). The r-value between the ERT and TIA is 0.76 (see Table 3). This shows that, in general, the ERT principles are significantly positively correlated and are related to the ability of teachers to integrate technology in learning (TIA). The two scales measure theoretically different constructs, supporting the discriminant validity inferred at step 5. Therefore, the validity and reliability tests validate the measurement model.

**Table 3:** Confirmatory composite analysis (CCA) results.

	Mean	S.D	SLF
Step 1–2			
Simplicity	4.20	0.79	0.84
Accessibility	4.16	0.87	0.83
Affordability	3.83	1.03	0.75
Flexibility	4.11	0.89	0.82
Empathy	4.10	0.84	0.83

Step 3–4			
Cronbach's Alpha (CA)	0.87		
Composite Reliability (CR)	0.91		
Average Variance Extracted (AVE)	0.66		

Step 5–6			
Correlation between TIA and ERTP	0.76		
Root square AVE	0.81		

Notes: ERTP = Emergency remote teaching principles, TIA = technology integration and application, S.D = standard deviation, SLF = standardized loading factor.

A summary of the collected data regarding the type of university (private-state university), gender, age, and employment is shown in Table 4. Approximately 68 percent of the participants are students from private universities, and 67.8 people are women. Regarding the employment status, 60.5 students were unemployed, and most respondents were under 25 years old (78%). Sixty-one percent of respondents come from the Jakarta Province, which 13 universities represent. Jakarta is the center of this survey because it is the capital city and has the best technology infrastructure than other locations.

**Table 4:** Demographic characteristics.

	freq.	Percent	Mean
University type			
Private University	2010	67.97	
State University	947	32.03	

Gender			
Female	2006	67.84	
Male	951	32.16	

Employment			
Employed	1168	39.50	
Unemployed	1789	60.50	

Age			
<25 yo	2311	78.15	
25–30 yo	374	12.65	
31–40 yo	173	5.85	
>41 yo	97	3.28	

(blank)	2	0.07	

**Figure 1:**
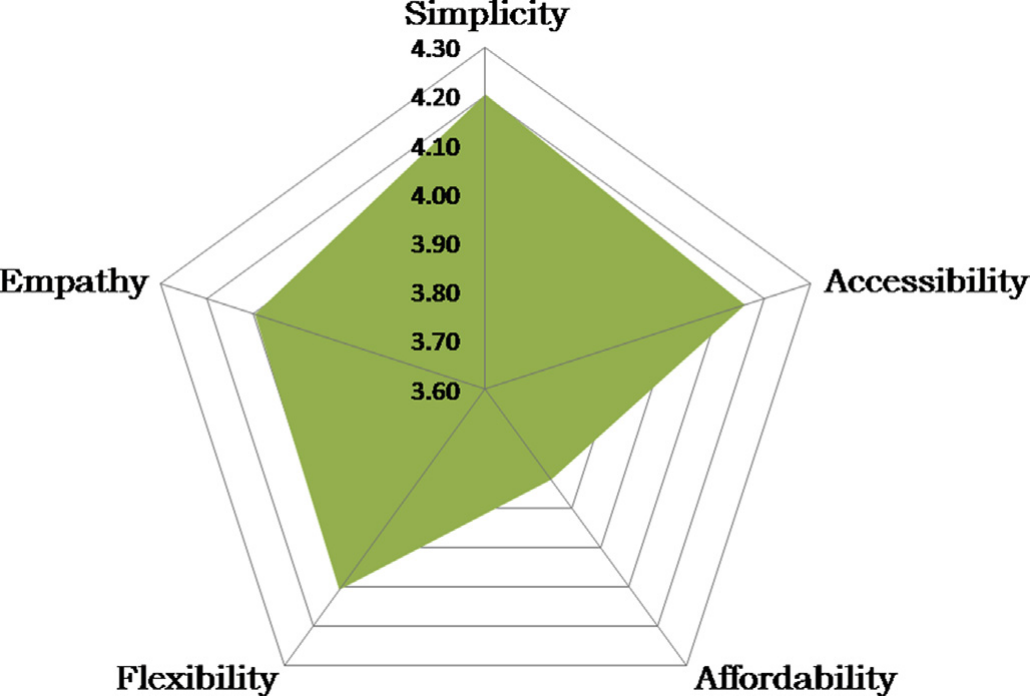
Mean rating of five principles of ERT (n = 2,957).

Figure 1 display the mean scores for perceived ERT principles. Of the five principles proposed, simplicity and accessibility received the highest ratings, followed by flexibility, empathy, and affordability.

## 5 Discussion

This study's main objectives are to explore the principles for implementing ERT and the challenges behind the implemented ERT model based on students' perceptions. For these purposes, two studies were conducted: the first study aimed to explore the relevant principles in implementing ERT involving an expert panel for content validity, readability, and logical flow. Content validity was analyzed to answer the first question quantitatively, and reflection reports were analyzed to answer the second question qualitatively. A different method was used to ensure that the principles developed had appropriate content and were relevant to Indonesia's conditions. The next phase, a qualitative endeavor involving administrators and faculty members to reflect on the proposed principle. Study 1 produced five main principles (simplicity, accessibility, affordability, flexibility, and empathy) for evaluating remote teaching activities.

Study 2 aims to test the reliability, validity, and ranking of the proposed ERT principles from Study 1. The broad geographical coverage in this study gaining a rich perspective on the implementation of ERT because, in general, there are disparities in technological infrastructure between regions and differences in Indonesia. Study 2 involved 2,957 undergraduate students from 22 universities in ten Indonesian provinces. This second stage study also provides an overview of student responses to the five principles generated in Study 1. The survey results show that the highest rating according to respondents' perceptions is simplicity and accessibility, followed by empathy, flexibility, and affordability.

### 5.1 Theoretical implications

From a theoretical perspective, this study has several contributions: first, these five ERT principles synthesize the three principles proposed by The University of Auckland (2020) such as simplicity, flexibility, and empathy, emergency curriculum design (Wang and East, 2020), and the critical work of Hodges et al. (2020) regarding the differences between ERT and online learning. It is not sufficient to base ERT solely on the three principles of simplicity, flexibility, and empathy. It is also necessary to consider two additional aspects, accessibility and affordability, based on community socioeconomic considerations and differences in the speed of Internet access, which often become obstacles, particularly in developing countries. Therefore, we propose five principles—simplicity, accessibility, affordability, flexibility, and empathy—by combining the previous works (Hodges et al., 2020; Mohmmed et al., 2020; The University of Auckland, 2020).

Second, this study offers universal principles for implementing ERT using online learning modes in various crises (e.g., natural disasters, wars, and other crises). Furthermore, this study involves a broad sample, covering ten provinces in Indonesia with different geographical and technological readiness. Thus, this study has a broader generalization, especially in Indonesia and developing countries with the same geographical characteristics.

**Figure 2:**
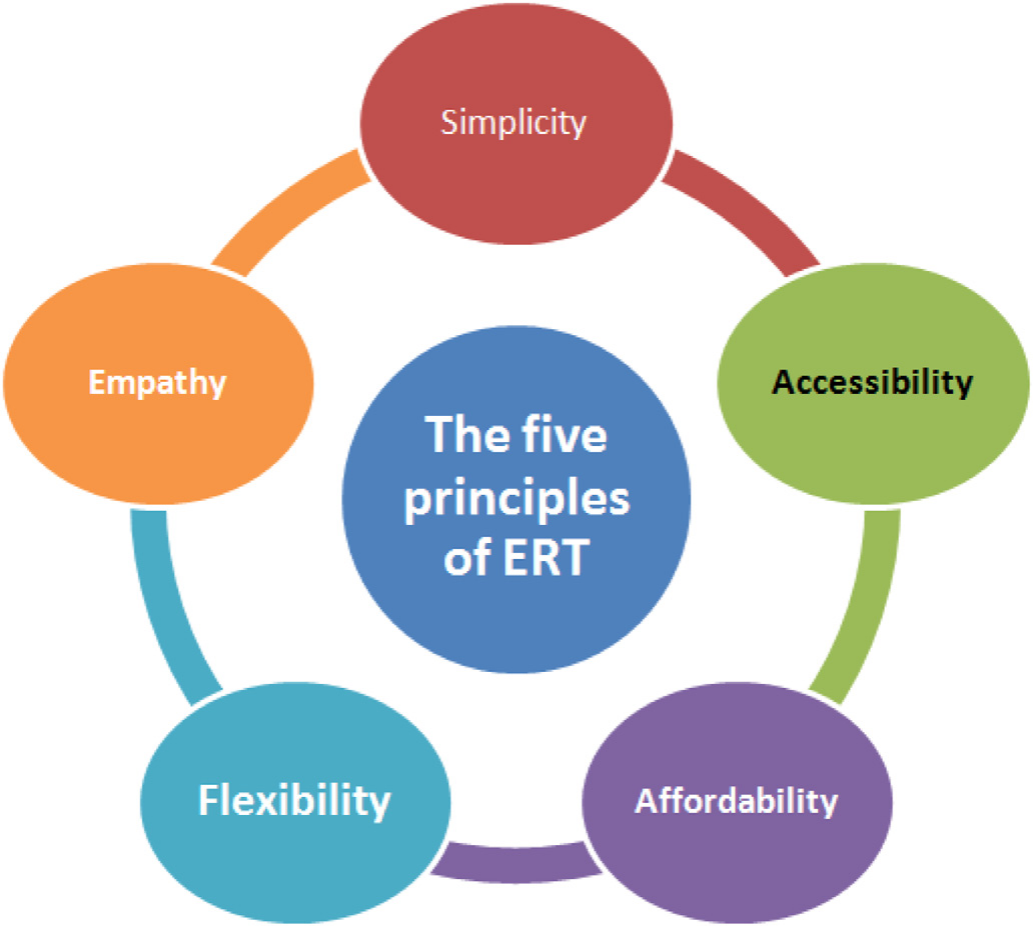
Five principles of emergency remote teaching.

Third, ERT principles cannot be separated from the psychological and socioeconomic aspects. In an emergency, we cannot obsessively focus on teaching delivery, knowledge transmission, and lecturing using sophisticated technology (Bozkurt and Sharma, 2020). We support the term “humanizing pedagogy” (Karakaya, 2020), in which the focus of ERT is to push beyond purely cognitive approaches and become more reflexive. Humanizing pedagogy as a part of the human-centered design may help university teachers to build empathy. Therefore, the ERT design needs to be preceded by assessing students’ needs and available technological resources. In general, these five principles support learning design based on evaluating needs and environmental situations (e.g., physical, psychological, economic, health, and spiritual). Thus, the five principles can be integrated with the ACAD framework (see Carvalho and Goodyear, 2018) and are in line with human-centered design (Salazar, 2013) (see Figure 2).

### 5.2 Practical implications

From a practical viewpoint, ERT should be treated as a temporary solution and should be distinct from online learning (Wang and East, 2020). In this sense, online learning and ERT are not the same; they differ in both purpose and function. Imposing teacher-selected or technology-driven learning delivery without considering the macro socioeconomic environment or individual resources could mean that ERT will not meet the principles of accessibility, simplicity, and affordability. For example, using special programs that require fast Internet access and high random-access memory (RAM) on a mobile device will cause new problems, such as obstruction of the learning process and increase quota consumption quickly. Furthermore, most Indonesian students rely on expensive limited-capacity mobile networks, making it challenging to meet their online learning requirements. Consequently, many technical problems can occur in video conferences, such as loss of sound, delayed images, or inability to access classes due to low-speed Internet networks or devices' technical capabilities (for example, the device may not meet the minimum requirements for the application). This condition causes new problems in the learning process rather than make it easier for students. This study argues that the implementation of ERT should be a collective decision by all participant groups. By conducting discussions with groups of students and lecturers, the most effective midpoint may be found to implement learning and reduce the effects of technological boundaries.

Schools/administrators need to understand that this is not a normal situation in which learning competency standards must be rigorously met. In a crisis, and given facts from the field that show disparities in technology and Internet networks, curriculum fulfillment is not the only issue of concern; it is also essential to care for and support learners during this difficult time. Thus, university administrators must ensure that teaching staff has two essential lecturer competencies, technical and pedagogical. To be effective, administrators should focus on the technical skills of lecturers to run ERT. Although this task can be assisted by IT support in some ways, all lecturers need to prepare technical knowledge and skills in managing online-based learning for the effectiveness of future learning. Apart from technical skills, the most critical aspect is pedagogical ability in managing learning. This ability is needed to maintain student motivation under challenging situations. The principle of ERT is to provide educational services that are simple, accessible, affordable, and flexible and provide precise support to students with an empathetic attitude rather than just delivering the best lectures.

Finally, the COVID-19 pandemic has become a stimulus and motivation for educational institutions to start investing in learning management systems (LMS) as an option to increase the ability to adapt to various unexpected situations in the future.

### 5.3 Limitations and future research

This study obtained opinions from teachers, administrators, and students at universities to capture the current situation and various inputs related to the five proposed principles. Some limitations of this study: first, the short duration of the study did not allow an in-depth evaluation of these principles. A longitudinal study should be performed to evaluate the principle in different situations and countries. Second, this study was conducted explicitly in Indonesia to describe the implementation of education in Indonesia during the COVID-19 pandemic. Thus, we invite researchers in other countries to add other principles that this study may not have identified. Future researchers can revisit the principles based on the technological readiness and socioeconomic conditions of each region. For example, a country with good quality technology infrastructure and low economic inequality is likely to focus more on simplicity, flexibility, and empathy than affordability and accessibility.

Third, this study focuses on the application of ERT principles in the context of online learning. We realize that ERT is not exclusive to online learning only, so that future studies need to expand the coverage area to conventional learning modes. Thus, the five principles constitute one unit whose order can be adjusted after considering the situation in the field. Finally, we did not consider the possibility of bias due to the unequal distribution of respondents, especially in Study 2, the majority of whom were women (67.84%), from private universities (67.97%), and unemployed (60.50%). Future studies can provide more attention to the distribution of respondents and the possibility of demographic bias.

## 6 Conclusions

This study provides basic principles for future emergency remote teaching implementation. However, since the primary focus of ERT is not on using sophisticated technology to transfer educational content over the Internet, but instead, it is a temporary solution- it should be designed based on simplicity, accessibility, affordability, flexibility, and empathy in all learning activities in unfavorable situations.

The lack of preparation to shifts conventional to online courses, differences in the level of readiness of internal resources (such as technical knowledge and skills in managing online-based learning), and the quality of technology infrastructure are common problems in major countries in the implementation of ERT. Therefore, learning design in ERT is dynamic and open to revision based on socio-economic considerations, technological infrastructure, and readiness for students and teachers. Specifically for developing countries with limited technical infrastructure and inequality in internet access, simplicity, accessibility, and affordability may be the primary concern, and in contrast, developed countries may focus more on simplicity, flexibility, and empathy.

Finally, the COVID-19 pandemic has made us realize that a robust information technology infrastructure is the key to the success of remote education. The speed needed to adapt to environmental changes requires high preparedness, and adaptation cannot proceed effectively without adequate technological resources. Furthermore, it is essential to increase the readiness of administrators, teachers, and students to switch from face-to-face instruction to an online mode based on experiences during the pandemic. These ERT experiences can increase the readiness of online learning after the pandemic, both teachers and students, forming new skills - working and school from home.

## Declarations

### Author contribution statement

Ani Cahyadi: Conceived and designed the experiments; Analyzed and interpreted the data; Wrote the paper.

Hendryadi: Conceived and designed the experiments; Performed the experiments; Analyzed and interpreted the data; Wrote the paper.

Sri Widyastuti, Achmadi: Performed the experiments; Contributed reagents, materials, analysis tools or data.

Vika Nurul Mufidah: Performed the experiments; Contributed reagents, materials, analysis tools or data; Wrote the paper.

### Funding statement

This research did not receive any specific grant from funding agencies in the public, commercial, or not-for-profit sectors.

### Data availability statement

Data associated with this study has been deposited at https://doi.org/10.5281/zenodo.4692674.

### Declaration of interests statement

The authors declare no conflict of interest.

### Additional information

No additional information is available for this paper.
